# Postpartum Invasive Group A Streptococcus Infection: Case Report and Mini-review

**DOI:** 10.7759/cureus.3184

**Published:** 2018-08-22

**Authors:** Michelle Nguyen, Venkata Sunil Bendi, Mounika Guduru, Evan Olson, Renuga Vivekanandan, Pamela A Foral, Manasa Velagapudi

**Affiliations:** 1 Creighton University School of Medicine, CHI Creighton University Medical Center, Omaha, USA; 2 Neurological Sciences, University of Nebraska Medical Center, Omaha, USA; 3 Surgery, Guthrie Robert Packer Hospital, Sayre, USA; 4 Infectious Disease, CHI Health, Creighton University, Omaha, USA; 5 Pharmacy Practice, Creighton University School of Pharmacy and Health Professions, Omaha, USA; 6 Infectious Diseases, Creighton University Medical Center, Omaha, USA

**Keywords:** group a strep, postpartum group a streptococcus, group a streptococcus, postpartum endometritis, streptococcus pyogenes, postpartum gas, postpartum gas infection, invasive group a strep, invasive gas disease, invasive gas infection

## Abstract

The overall incidence of postpartum invasive group A streptococcal (GAS) disease is low in the United States. However, postpartum women are much more likely to develop GAS disease than nonpregnant women. Additionally, postpartum GAS has the potential to develop into a severe disease and a delay in diagnosis can have deadly consequences. This case describes a patient with invasive postpartum endometritis in the setting of diastases of the pubic symphysis. Sepsis secondary to the endometritis develops along with bilateral pneumonia. This case characterizes some of the typical and atypical symptoms a patient with invasive postpartum GAS can present with. Further, it outlines the timely identification of the disease and its appropriate treatment to prevent a potentially disastrous outcome.

## Introduction

Group A streptococcal (GAS) disease risk is significantly higher among postpartum women compared with non-pregnant women, with the risk being 20 times higher amongst pregnant women [1]. However, the overall incidence is low, with only 220 cases of postpartum invasive GAS infection being reported annually in the United States from 1995-2000 [2]. The identification of GAS infections during pregnancy or in the postpartum period can be challenging due to the rarity of the disease and its varied presentation. While GAS disease can be non-invasive, it can also cause severe invasive illnesses like septicemia, toxic shock syndrome (TSS), and necrotizing fasciitis. Delay in the diagnosis of invasive GAS can have disastrous consequences due to the aggressive course of the disease. For example, once shock develops, mortality approaches 40-60% [1].

## Case presentation

**Figure 1:**
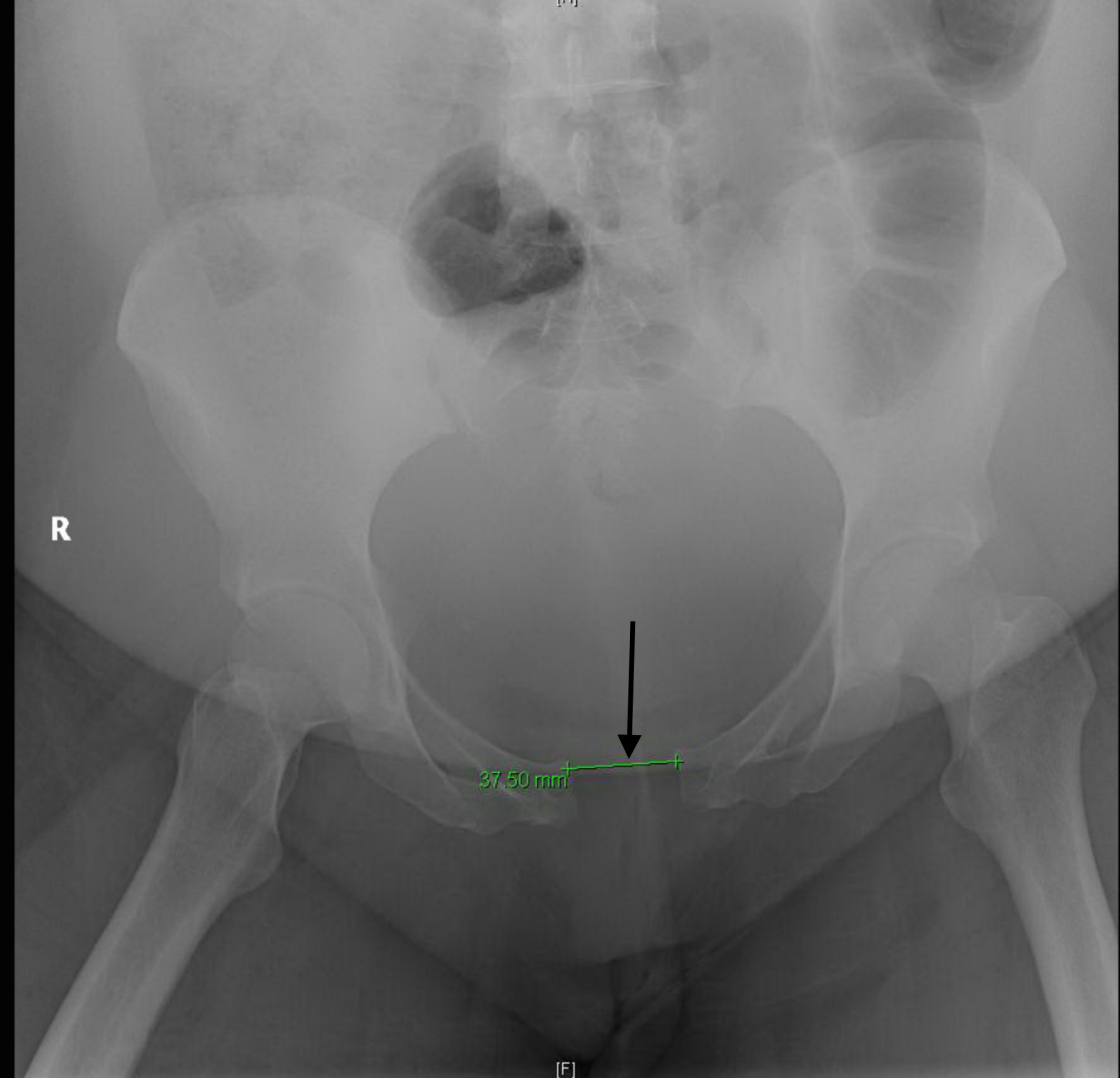
Pelvic X-ray showing diastases of the pubic symphysis measuring 3.75 cm

A 32-year-old obese woman gravida five, para 3104, with a history of preterm contractions and preeclampsia, presented to the emergency department with persistent pelvic pain and a fever of 103.1 F after delivering a baby girl four days prior, via a normal spontaneous vaginal delivery at term. Per the patient report, she had been experiencing fever and chills since the delivery. On physical exam, she was found to have a normal amount of foul-smelling lochia. The labs showed an elevated white blood cell (WBC) count and anemia. A pelvic x-ray showed diastases of the pubic symphysis measuring 3.75 cm (Figure 1). A magnetic resonance imaging (MRI) scan of the pelvis and lumbar spine failed to show a pelvic abscess or hematoma.

The patient was admitted for further evaluation. Over the course of the day, the patient became progressively tachycardic with a fever of 102.5 F, and sepsis secondary to the endometritis was suspected. The patient was started on intravenous (IV) ampicillin and clindamycin. The following day, blood cultures were drawn showing *Streptococcus pyogenes* (group A) and *Proteus mirabilis*. A urine culture grew 80,000 cfu/mL of *Proteus mirabilis*. The *Proteus mirabilis* was susceptible to ampicillin, cefazolin, ceftriaxone, ciprofloxacin, gentamicin, levofloxacin, and trimethoprim/sulfamethoxazole and resistant to nitrofurantoin. Susceptibility for GAS was not done, as routine susceptibility for beta-hemolytic streptococcus was not routinely performed at the institution due to its usual susceptibility to the penicillin family.

**Figure 2:**
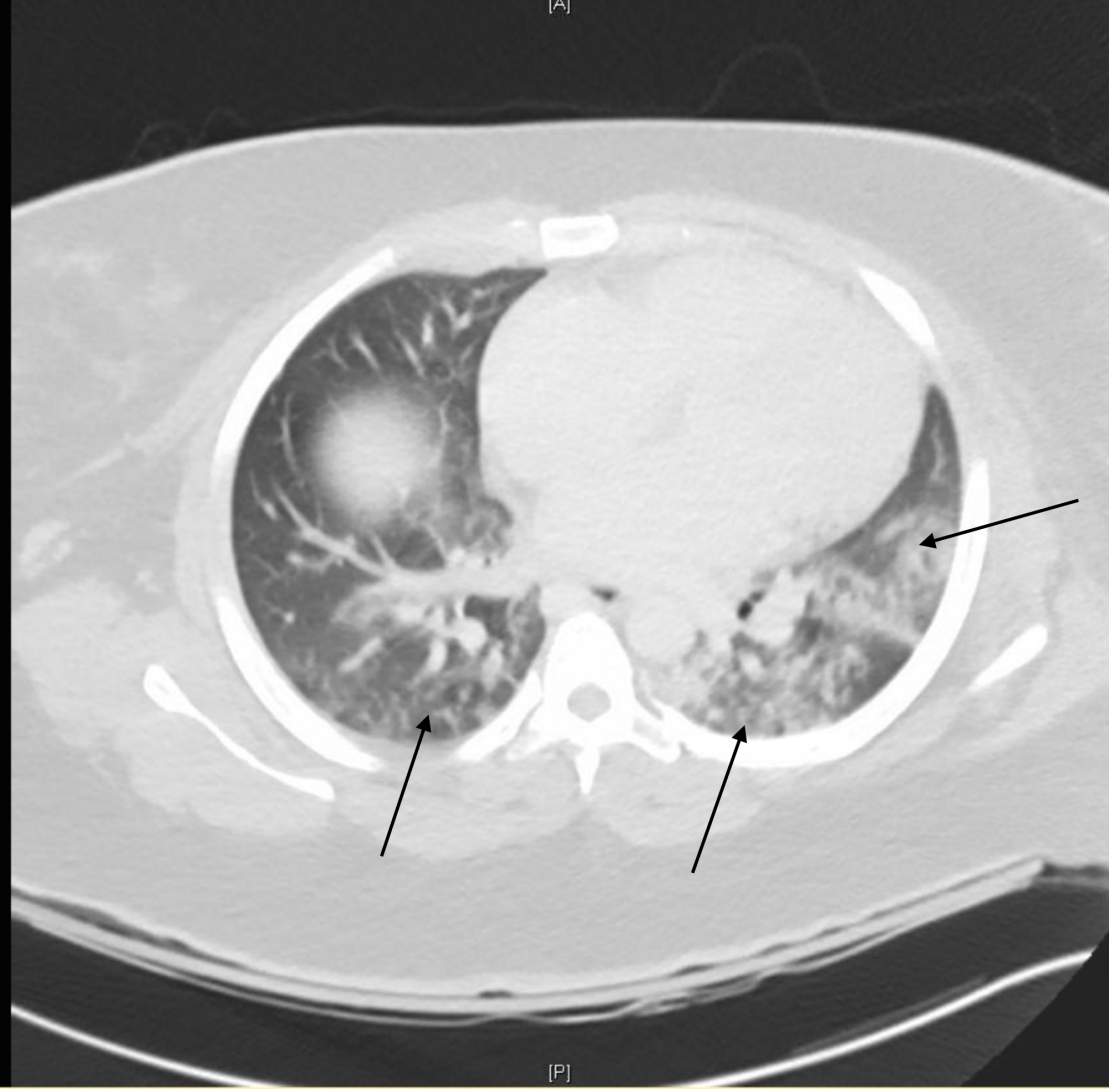
Computed tomography (CT) scan of the chest showing bilateral patchy opacities

On hospital day three, the patient was noted to be febrile, tachypneic, tachycardic, and hypoxic. She was also experiencing a productive cough. Expiratory wheezes were heard in the upper lung fields bilaterally. A chest computed tomography (CT) showed bilateral patchy opacities (Figure 2). Hospital-acquired pneumonia was diagnosed, based on the patient's clinical picture and CT scan. Seven days of empirical therapy for hospital-acquired pneumonia was started, which included a one-time dose of azithromycin and a course of IV piperacillin/tazobactam, vancomycin, and levofloxacin. Repeat blood cultures that same day showed no growth. On hospital day eight, vancomycin was discontinued as there was no evidence of methicillin-resistant *Staphylococcus aureus* (MRSA) in the blood or nares culture. The antibiotics were switched to two grams of IV ceftriaxone once a day to treat the GAS endometritis with bacteremia. Oral metronidazole, 500 mg every eight hours, was also added for empirical anaerobic coverage. Ceftriaxone was chosen over penicillin, given the convenience of once a day dosing with ceftriaxone as compared to penicillin. She improved clinically, became asymptomatic, and was discharged to a skilled nursing care facility. At her three-week outpatient follow-up visit, the patient was doing well. She had completed a four-week course of IV ceftriaxone and oral metronidazole, and an additional course of amoxicillin/clavulanate was initiated for two more weeks.

## Discussion

Epidemiology

In the past, outbreaks of postpartum GAS infections have been associated with transmission from healthcare workers [3]. In the mid-19th century, Semmelweis made the groundbreaking discovery that physicians were transmitting “childbed fever” from their hands to pregnant women during labor and delivery. It was later discovered that *Streptococcus pyogenes* was the causative agent for childbed fever in Semmelweis’ clinic [1].

In six out of the 10 outbreaks of postpartum infections reported since 1965, healthcare workers who were the asymptomatic carriers of GAS were identified, but the proportion of cases of invasive postpartum GAS infection attributable to infected healthcare workers is unknown [3]. Investigation of these cases suggests that the prompt institution of investigation and control measures may prevent further postpartum morbidity and the establishment of an appropriate epidemiologic investigation may assist in these preventative efforts [3].

Today, most cases of pregnancy-associated GAS infection are community acquired, with only 15%-25% being nosocomial [1]. The cause of the infection may be the ascension of the organism from the maternal vagina, as well as transmission from infected or colonized contacts. In the United States, the annual incidence of GAS postpartum infections is reportedly low, despite a 20-fold increased risk among postpartum women compared to non-pregnant women [1].

In a population-based surveillance for GAS infections during pregnancy or in the postpartum period by Chuang et al., it was reported that 85% of GAS occurs postpartum, with most occurring after vaginal delivery, within the first four days after delivery [3]. Similarly, in a review of published cases and case series of postpartum GAS infections from 1974-2009, it was reported that 84.4% of GAS infections followed vaginal delivery, and that 72.5% occurred within the ﬁrst four days postpartum [2]. It was estimated that 220 cases occurred among postpartum women annually in the United States [3]. Of these, 64% of patients were Caucasian and 28% were African American. The majority of the infections were bacteremia (46%), endometritis (28%), peritonitis (8%), and septic abortion (7%).

Pathophysiology

Hamilton et al. [2] suggest that the increased susceptibility to GAS infection in pregnant women compared to non-pregnant women could be due to compromised mucosal or cutaneous barriers, change in vaginal pH after amniotic fluid release, or suppressed innate immunity.

It has been shown that immunologic polymorphisms contribute to the susceptibility to GAS infection in general, though no studies have specifically investigated this relative to postpartum GAS infections. A much larger population of women are colonized with GAS than those actually developing an infection, which suggests either an inherent resistance to GAS or that not all colonizing strains of GAS are capable of causing an infection [2].

A major virulence factor of GAS includes the M protein. Strains with a large number of M protein are resistant to phagocytosis. Among postpartum GAS blood isolates, the most common M type is M28. A similar report of 18 cases from Utah demonstrated that severe GAS puerperal infection was solely associated with M types M1 and M28 [2].

Other virulence factors of GAS include toxins that stimulate T-cell production of inflammatory cytokines including interleukin-1B and tumor necrosis factor-a, which can lead to profound hypotension and diffuse capillary leaking. These toxins are also thought to cause liquefaction of purulent material allowing the spread of bacteria. Proteases produced by GAS release bradykinin, a potent systemic vasodilator that can lead to widespread organ failure [1].

Clinical features

Postpartum GAS infections tend to be difficult to identify not only because of their rarity, but also because of the variety of ways in which it can present. The majority of GAS infections occur within four days of delivery [4]. Though GAS is not a common cause of postpartum endometritis, it can still present in the typical fashion with fever, uterine pain, and malodorous vaginal discharge [2]. In contrast, initial symptoms and signs may be mild and nonspecific. Nonspecific viral symptoms, such as fever, chills, nausea, vomiting, and myalgia have been reported by 20% of patients. Fever usually exceeds 102 F, which is unusual in typical cases of postpartum endometritis [4]. Other atypical signs and symptoms include dyspnea, rash, pharyngitis, headache, and confusion or combativeness. These symptoms may occur in the absence of fever and may be the only indication of GAS [2].

Physical signs like unilateral or bilateral lower abdominal tenderness may be minimal and do not correspond to the severity of the infection. Other signs can include foul-smelling or scanty to non-purulent odorless lochia, which is usually the case with a postpartum GAS infection. On the contrary, some may present with severe pain and tenderness in the pelvic region and marked leukocytosis [4].

Postpartum females with minimal pelvic tenderness and absence of fever are prone to develop sepsis due to delay in diagnosis, especially when endometrial sampling is difficult in the early postpartum period [2]. Septicemia may result in septic shock and multiorgan failure, which are fatal conditions until treated appropriately. It is important to differentiate TSS from sepsis by evaluating liver and kidney function. Both are invasive complications of post-partum GAS endometritis; however, TSS is responsible for early renal insufficiency and sepsis is responsible for late renal insufficiency [5].

Necrotizing fasciitis is a rare complication of a postpartum GAS infection, but it is highly deadly. Skin changes similar to cellulitis can be seen early on. Patients often become septic before marked skin changes like bullae and skin necrosis occur. Severe local pain out of proportion to the observed progression of erythema and edema is common [4].

Diagnosis

Because of the potentially aggressive course of GAS disease and the challenges in identifying it, an early diagnosis is key for preventing mortality [1]. GAS disease can be categorized into invasive GAS disease or toxic shock syndrome. Both diagnoses involve positive blood cultures or the isolation of GAS from a sterile site [3]. Invasive GAS disease may progress to TSS. The clinical case definition for TSS includes hypotension (sBP <90 mmHg), multiorgan involvement, generalized erythematous macular rash that may desquamate, and soft tissue necrosis, including necrotizing fasciitis or myositis, or gangrene [1].

Recognizing the clinical features of GAS disease characterized above is key towards diagnosis. GAS should be routinely included on the differential diagnosis in anyone presenting with atypical signs and symptoms during pregnancy or in the postpartum period [1]. Other warning signs to look for come from maternal early warning criteria, which suggests a serious risk of morbidity and mortality for the patient. These include: systolic blood pressure (BP) < 90 or > 160 mmHg, diastolic BP > 100, HR <50 or > 120 bpm, respiratory rate (RR) < 10 or > 30 breaths per minute, oxygen saturation on room air and at sea level < 95%, oliguria < 35 mL/hr for two hours, maternal agitation, confusion, unresponsiveness, and a patient with preeclampsia accompanied by a non-remitting headache or shortness of breath [6].

In addition to recognizing the signs and symptoms of GAS disease, lab abnormalities should also be noted, as they may be present in a patient who does not appear ill. A complete blood count often demonstrates leukocytosis, which can be a normal finding in postpartum women. An increased neutrophil count with bandemia and an elevated lactic acid concentration postpartum, are indicative of infection. Marked bandemia (greater than 10%) may be seen in the absence of leukocytosis [1].

If GAS disease is suspected, it is important to identify the source of infection and isolate the organism. Blood and urine cultures should be obtained to confirm GAS. Endometrial aspiration for a gram stain and culture should be performed if the uterus is the suspected source of infection. The presence of neutrophils and plasma cells confirms the diagnosis of endometritis [1]. Imaging modalities, such as CT, MRI, and ultrasound will typically demonstrate an edematous uterus larger than expected; however, they may appear normal and should not delay aggressive management. A CT scan is an effective tool in the work-up of a toxic patient in acute pain. Besides identifying sites of infection, it can disclose or rule out other causes of sepsis [1,5].

Treatment

Perioperative antibiotics have significantly decreased the incidence of endometritis. Though rare, due to the advanced development in the field of medicine, an invasive GAS infection may be a fatal condition in the postpartum period [7]. Antibacterial agents, antipyretics, and supportive therapy are key in the treatment of postpartum endometritis, and the outcome depends on the timing and the clinical severity of the illness. Education about proper perineum-cleaning techniques and nutritional care to all postpartum females would aid in wound healing.

Penicillin stands as the mainstay in treating postpartum endometritis and septicemia, as GAS is highly sensitive to it. The recommended dose is 4 million units every four hours, of intravenous penicillin-g [4]. The efficacy of penicillin against GAS is mainly dependent on the phase and concentration of the organism. A loss of penicillin-binding proteins (PBPs) in the stationary-phase growth and a higher concentration of microorganism contribute to the failure of penicillin. Hence, it is recommended to add another antimicrobial agent like clindamycin, whose efficacy remains unaffected by inoculum size or stage of growth [8]. Besides suppressing the synthesis of PBPs, clindamycin’s efficacy is more related to its ability to suppress the synthesis of M protein and bacterial exotoxins. It has also been shown to inhibit bacterial toxin synthesis and promotes phagocytosis [8].

In a combination therapy of clindamycin and ampicillin, ampicillin can be replaced by cefazolin or vancomycin in patients who are allergic and anaphylactic to penicillin, respectively [4]. Toxic shock syndrome (TSS) is a complication of invasive GAS infection and may lead to death if not treated appropriately. Though immune globulin has no benefit in invasive GAS infections, it shows dramatic clinical improvement in TSS [9]. The presence of TSS even after prompt treatment for invasive GAS infection is an indication for hysterectomy, where reversal of the disease course occurs with the removal of the source of GAS (i.e. the endometrium) [9].

Prognosis and outcomes

In the previously mentioned population-based case series by Chuang et al., the case fatality rate for invasive postpartum GAS disease among infected women (3.5%) was lower than that for other invasive GAS infections (9.4%) among women aged 10–50 years [3]. In a population-based surveillance for invasive GAS from 2000-2004, the overall rate of invasive GAS was 3.48 per 100,000 persons with a case-fatality rate of 13.7%. Of the cases of invasive GAS identified, only 2.6% had recently been pregnant and had a 4.3% case fatality rate [1].

Data from the Centers for Disease Control and Prevention (CDC) report the occurrence of approximately 10,000 cases of invasive disease each year in the United States, with death occurring in 10%-15% of these cases. Once shock develops, however, mortality reaches 40%-60% [1].

Fetal outcomes were reported as follows: 60 infants (82%) did not develop any apparent illness, four infants (5%) had a nonspecific clinical disease, seven pregnancies (10%) resulted in septic abortions or stillbirth, and two pregnancies (3%) resulted in an induced abortion. No neonatal deaths were reported [3].

Several epidemiological, host, and microbial factors contribute to the risk of GAS infections and mortality in postpartum women. These include the mode of delivery (vaginal versus Cesarean section), the location where labor and delivery occurred, exposure to GAS carriers, the altered immune status associated with pregnancy, the genetic background of the host, the virulence of the infecting GAS strain, and highly specialized immune responses associated with female reproductive tract tissues and organs [10]. The factors that affected outcome were the timing of the onset of the GAS infection in relation to the pregnancy and delivery and emm type [2]. GAS infection during the third trimester of pregnancy resulted in grave outcomes to mother and fetus. Infections five to eight days postpartum have greater systemic involvement with possible hematogenous seeding of the extremities, while late postpartum infections resulted in 100% maternal survival and no infant mortality [2].

## Conclusions

Despite the low incidence of GAS disease in pregnant women, this population still remains at a significantly increased risk for GAS disease compared to non-pregnant women. GAS disease in the postpartum period can be difficult to identify due to its atypical presentation. Postpartum GAS can quickly progress to invasive disease and delayed diagnosis can be fatal. With the use of perioperative antibiotics, the incidence of postpartum invasive GAS has decreased. The cornerstone of treatment consists of antibacterial agents and supportive therapy with the outcome being dependent on the timing and the clinical severity of the illness. This case report is an excellent example of how postpartum GAS can quickly become invasive, the atypical symptoms with which invasive disease can present, and the timely and appropriate identification and treatment of the disease.
